# Mycetoma by Actinomadura madurae in the central nervous system: Renal transplant receptor disease

**DOI:** 10.1002/ccr3.6071

**Published:** 2022-07-25

**Authors:** José Ignacio Cerrillos‐Gutiérrez, Diana Ramírez‐Flores, Alfredo Gutiérrez‐Govea, Jorge Andrade‐Sierra, Miguel Medina‐Pérez, Enrique Rojas‐Campos

**Affiliations:** ^1^ Department of Nephrology and Organ Transplant Unit, Specialties Hospital, National Western Medical Centre Mexican Institute of Social Security Guadalajara Jalisco Mexico; ^2^ University Health Sciences Center University of Guadalajara Guadalajara Jalisco Mexico; ^3^ Medical Research Unit in Renal Diseases, Specialties Hospital, National Western Medical Centre Mexican Institute of Social Security Guadalajara Jalisco Mexico

**Keywords:** abscess, mycetoma, nephrology, renal transplant

## Abstract

The mycetoma is a granulomatous chronic disease, subcutaneous disease is the common presentation, very few cases are reported affecting central nervous system, but there are not cases in Renal Transplant (RT).

## Graphical abstract

Renal transplant recipients have increased risk of invasive fungal infections. Transplant centers in geographic endemic risk areas should suspect the possibility of mycetoma infection in patients. This clinical entity is not commonly seen in the United States, and hence few cases have been reported.

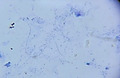

## 1 INTRODUCTION

Mycetoma is a granulomatous chronic disease that more frequently affects subcutaneous tissue^1^; there are only a few cases affecting the central nervous system (less than 5%).^2^ The disease is considered endemic in Mexico, with 3933 cases recorded in 54 years (8% caused by *Actinomadura Madurae*).^3^ Four cases of actinomycosis in renal transplant (RT) have been reported,^4^ but none of them are related to the central nervous system.

Mycetoma appears mostly in males than in females (3:1) and young adults might also be infected (16–40 years old). Lesions are often associated with trauma. Around 75% of mycetoma is thought to occur in the lower extremity. The incubation period can be found up to 9 years.^5^ Neurological manifestations are not presented usually, and the spread of infection to the brain is unknown.

## 2 CLINICAL CASE

A 25‐year‐old male, construction worker in Zapopan, Jalisco, Mexico, with a chronic kidney disease for 7 years was diagnosed with unknown cause. The patient received a renal graft in January 2017, from a related donor (sister): 35 years old, high risk to cytomegalovirus (CMV; D+; R−), intermediate risk to Epstein Barr virus (D+; R+), cross match negative; ABO compatible (A+); and sharing three class I antigens (A*23, A*24, and B*57) and four class II antigens (DRB1*04, DRB1*11, DQB1*03:02, and DQB1*06:01). Graft (left) has one artery and one vein, warm ischemia time is 1 min 8 s, cold ischemia time is 30 min; mean arterial pressure at clamping is 100 mmHg and after unclamping 105 mmHg, double J catheter was not required. No incidents were reported during RT. After treatment with basiliximab, the maintenance immunosuppressive therapy was tacrolimus (2 mg twice a day [BID]), mycophenolic acid (1 gram BID), and prednisone (5 mg per day). Prophylaxis to CMV valganciclovir 450 mg BID was given; CMV disease (gastrointestinal manifestation) was developed (8 months after RT), polymerase chain reaction (PCR) 73,400 copies/ml. Valganciclovir was re‐started 900 mg BID until no replication.

**FIGURE 1:**
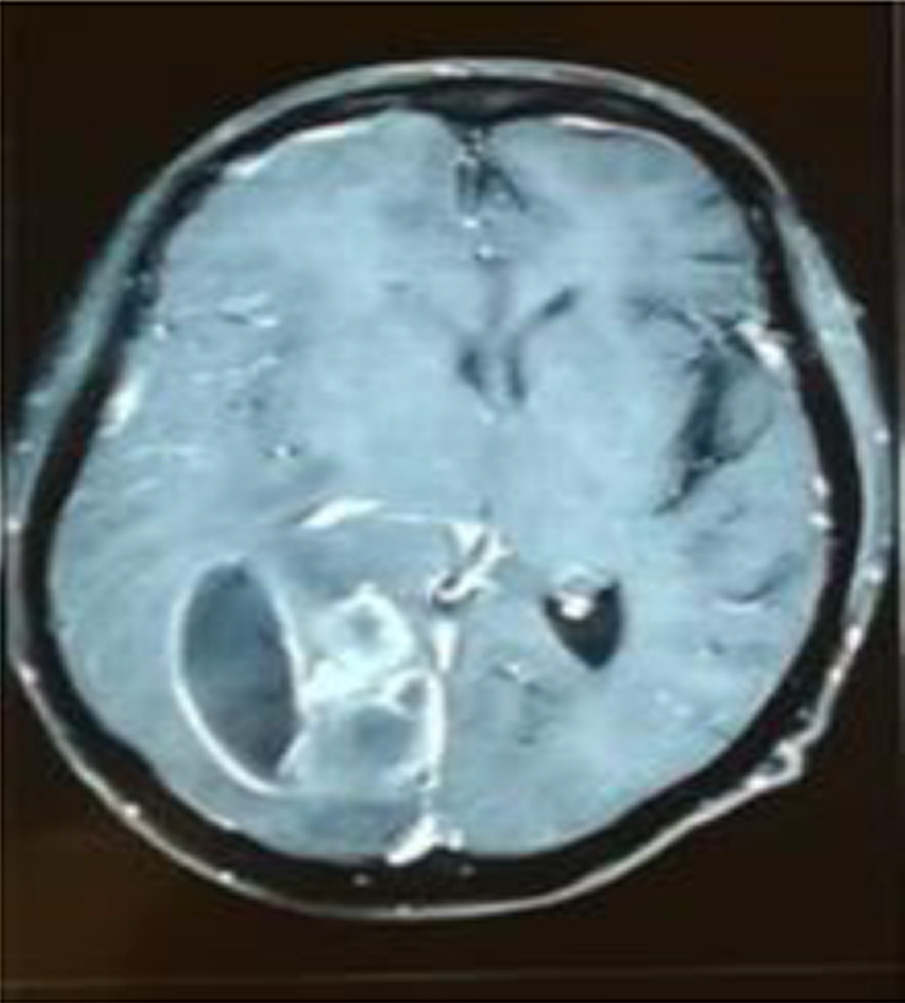
CT SCAN: Effacement furrows and brain fissures in the right temporal–parietal area. Homogeneous hypodense intra‐parenquimatous area, no vascular compromise is visible, with deviation of the midline structures and compressive effect

**FIGURE 2:**
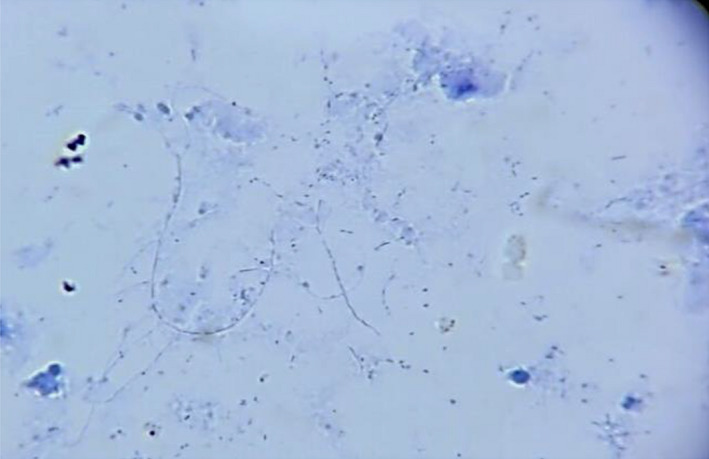
Culture in Saboraud, Micosel, and Lowenstein‐Jensen media, growing white‐yellow smooth, creamy colonies. Branched and delicate filaments can be found. The final observation suggested was *Actynomadura Madurae*

Ten months after RT, the patient refers having headache (it was associated with intracranial hypertension), no fever or neurological alterations were found; leucocytes were 3010 mm/μl, neutrophils 68% (2050 mm/μl), LDH 729 μ/L, and procalcitonin 0.30 ng/ml; computed tomography (CT) scan showed an abscess in the right parietal–temporal area (Figure 1), which was studied by puncture, and the results showed gram‐positive microorganisms. Metronidazole and cephtriaxone and trimethoprim/sulfamethoxazole were prescribed for 10 days (nocardia infection suspected). The mycological study (KOH and Lugol) revealed on direct examination grains comprising branched and delicate filaments. Microbiological culture media (Lowenstein‐Jensen media and Saboraud‐dextrose agar) showed creamy white‐yellow colonies, smooth consistency: *Actinomadura madurae* was identified, and then nucleotide sequencing of 16S rRNA PCR analysis was realized after 3 weeks of growth, at 37°C, it was established a certain diagnosis (Figure 2). Trimethoprim/sulfamethoxazole was prescribed for 1 year. A specific clinical re‐evaluation was done to find any abscesses, skin scratches, fistulas, nodules, etc., with unsuccessful results. The patient was discharged without cognitive, sensitive, and motor compromises. After 5 days of discharge, the patient complained of recurring headache. Cranial hypertension made us suspect reinfection and a new CT scan was solicited. The CT scan showed residual abscess and an asymmetric hydrocephaly: surgical draining and bypass valve were the treatment of choice. During the following month, the patient required two re‐admission events due to a mechanical valve compromise. The evolution of the renal graft was adequate, and the serum creatinine did not increase above 0.81 mg/dl, maintaining tacrolimus levels similar to that observed in other RT subjects without the illness.

## 3 DISCUSSION

Mycetoma is a chronic granulomatous disease usually that affects bone, subcutaneous, and deep tissues. There have been some reports that mention the infection could be disseminated. This pathology has recently been classified into a neglected disease, commonly observed in poor, low‐income countries.^6^

The patient is a rural community worker,^7^ who lived in close contact with livestock,^8^ in Jalisco, Mexico, an endemic region of *Actinomadura madurae*.^9^ Male gender has been usually associated with mycetoma caused by Nocardia Brasiliensis. *Actinomadura madurae* has been associated with females most commonly.^10^, ^11^

The patient did not have any clinical evidence for mycetoma infection. After first evaluation, we searched for any skin alteration without success. The mycetoma infection has an initial asymptomatic evolution. The final stage is characterized by pain.^1^ Cephalea due to cranial hypertension was the cause of hospitalization.

In vitro, the susceptibility of *A. madurae* to TMP/SMX is high, as is the efficacy in clinical practice. However, the treatment of actinomycetoma in most cases is prolonged, with complications, often expensive or with adverse events.^6^

Mycetoma caused by Actinomadura *madurae* is characterized by a continuous secretion,^1^, ^10^ suspected by the development of abscess. A bypass valve was needed to treat hydrocephaly.

Graft function was stable during the evolution. Tacrolimus levels were low during the evaluation. During infection, it was not necessary to decrease the tacrolimus dose.

This clinical case report is focused on the apparition of mycetoma in the central nervous system. No clinical manifestation of a kidney graft receptor was found during the illness. We found *Actynomadura madurae* to be the initial causative factor in immunocompromised patients. Transplant centers should suspect the possibility of mycetoma infection in patients, specially those who lives in geographic endemic risk areas, middle age group and farmers, as well, complete clinical evaluations to find mycetoma‐related skin lesions.

## AUTHOR CONTRIBUTIONS

CG was involved in primarily treating clinicians, reviewing the manuscript and final approval. AS and MP were involved in treating clinicians. RC was involved in coordinating case selection, reviewing the draft of the paper, interpreting data and final approval. RF was involved in drafting the manuscript. GG was involved in drafting the paper, creating a data set and descriptions for the original draft, revisions, and final approval.

## CONFLICT OF INTEREST

The authors declare no conflicts of interest associated with this manuscript.

## ETHICAL APPROVAL

As a single case report with the patient's signed consent, no other ethical review was required.

## CONSENT

Written informed consent was obtained from the patient to publish this report in accordance with the journal's patient consent policy.

## Data Availability

All basic clinical data have been reported. Physiological assessment data for all treatments and diagnoses are available upon request from the corresponding author.
